# Time course of brain activity during the processing of motor- and vision-related abstract concepts: flexibility and task dependency

**DOI:** 10.1007/s00426-020-01374-5

**Published:** 2020-07-13

**Authors:** Marcel Harpaintner, Natalie M. Trumpp, Markus Kiefer

**Affiliations:** Section for Cognitive Electrophysiology, Department of Psychiatry, Ulm University, Leimgrubenweg 12, 89075 Ulm, Germany

## Abstract

Grounded cognition theories assume that conceptual processing depends on modality-specific brain systems in a context-dependent fashion. Although the relation of abstract concepts to modality-specific systems is less obvious than for concrete concepts, recent behavioral and neuroimaging studies indicated a foundation of abstract concepts in vision and action. However, due to their poor temporal resolution, neuroimaging studies cannot determine whether sensorimotor activity reflects rapid access to conceptual information or later conceptual processes. The present study therefore assessed the time course of abstract concept processing using event-related potentials (ERPs) and compared ERP responses to abstract concepts with a strong relation to vision or action. We tested whether possible ERP effects to abstract word categories would emerge in early or in later time windows and whether these effects would depend on the depth of the conceptual task. In Experiment 1, a shallow lexical decision task, early feature-specific effects starting at 178 ms were revealed, but later effects beyond 300 ms were also observed. In Experiment 2, a deep conceptual decision task, feature-specific effects with an onset of 22 ms were obtained, but effects again extended beyond 300 ms. In congruency with earlier neuroimaging work, the present feature-specific ERP effects suggest a grounding of abstract concepts in modal brain systems. The presence of early and late feature-specific effects indicates that sensorimotor activity observed in neuroimaging experiments may reflect both rapid conceptual and later post-conceptual processing. Results furthermore suggest that a deep conceptual task accelerates access to conceptual sensorimotor features, thereby demonstrating conceptual flexibility.

## Introduction

What is the meaning of the concept “beauty,” a concept, which can be considered as being abstract as it does not refer to a physical referent? Philosophers have tried to define the meaning of this concept for several centuries based on more or less complex considerations (e.g., Baumgarten, 1750–1758). Imagining a tour through the Museum of Modern Art in New York probably makes these considerations much simpler. Imagine standing in front of the Starry Night painted by post-impressionist Vincent van Gogh and admiring the wavelike forms and the color gradient of the nocturne painting. Intuitively, most people would speak of beauty here. This example illustrates that, similar to concrete concepts such as “painting,” the meaning of abstract concepts might be related to perception (e.g., vision) through their reference to situations as suggested by grounded (or embodied) cognition theories. Thinking about the abstract concept “fitness” illustrates another notion of grounded cognition theories that (abstract) concept meaning might not only be grounded through its relation to (visual) perception but also through actions, such as lifting weights, doing yoga or bicycling (for reviews see Barsalou, 2008, 2010; Kiefer & Barsalou, 2013; Kiefer & Pulvermüller, 2012; Meteyard, Cuadrado, Bahrami, & Vigliocco, 2012; Pulvermüller, 2013).

Whereas it is generally accepted that concepts are the basic units of cognition that make up the meaning of words (Humphreys, Riddoch, & Quinlan, 1988; Kiefer & Pulvermüller, 2012; Tulving, 1972), their representational format and their organization at a neural and functional level are still a matter of debate. Traditional amodal approaches (Anderson, 1978; Collins & Loftus, 1975; Fodor, 2001; Mahon & Caramazza, 2009; Pylyshyn, 1980) would answer these questions by amodal semantic hubs, in which all forms of conceptual knowledge are represented (Rogers et al., 2004). In amodal models, the representational format of concepts is considered abstract and independent of motor interactions or perceptual impressions during concept acquisition. Original modality-specific experiential information is transformed into a common symbolic code (Caramazza & Mahon, 2003). Concrete and abstract concepts like “painting,” “beauty” or “fitness” are seen to be similarly represented separated from the sensorimotor brain systems in the same amodal code. Considering “beauty” from the beginning of this article, traditional amodal theories would assume that experiences gathered by the visitor of the Museum of Modern Art do not contribute to its representation of the concept “beauty,” since the original experiential information is transformed into an abstract code and is therefore seen to be lost or at least extenuated (Anderson, 1978; Caramazza & Mahon, 2003; McClelland & Rogers, 2003). Anterior inferior-temporal (Patterson, Nestor, & Rogers, 2007; Visser, Jefferies, & Ralph, 2010), inferior-parietal (Binder & Desai, 2011), posterior middle temporal (Gold et al., 2006; Hoffman, Pobric, Drakesmith, & Ralph, 2012; Price, 2000) and prefrontal cortex (Devlin, Matthews, & Rushworth, 2003) have been proposed as neural basis of these amodal hub regions, even though their exact number, function and location remain questionable (Meteyard et al., 2012). Activation of modal brain areas during conceptual processing is not denied per se; however, their engagement is considered, if at all, as epiphenomenon, which is not causally involved in conceptual representation: Activation of modal brain areas may occur concomitantly through spreading activation (Mahon, 2015a, 2015b) or strategic imagery (Machery, 2007), after a putatively amodal concept had been accessed.

Grounded cognition theories, in contrast, consider modal brain systems and interconnected networks including regions associated with motor, sensory, emotional and introspective processes as essential for conceptual representation (Barca, Borghi, Dove, & Tummolini, this issue; Barsalou, 2008; Borghi et al., 2017; Ghio, Vaghi, Perani, & Tettamanti, 2016; Kiefer & Barsalou, 2013; Pulvermüller & Fadiga, 2010). Concepts are seen as simulations of previous experiences and implemented in distributed brain networks, which arise from simultaneous activation of cell assemblies during concept acquisition (Pulvermüller & Fadiga, 2010), similarly as it already has been proposed by Hebbian theory (1949). Considering the examples of the beginning of this article, grounded cognition approaches would assume that the representational format of “beauty” or “fitness” is essentially related to the original perceptual impressions during concept acquisition like specific visual (e.g., forms and colors in the Starry Night) and motor (e.g., motor sequence of weight lifting and bicycling) information. Note that mental simulations based on modal representations are not necessarily accompanied by conscious experiences such as imagery (Kiefer & Barsalou, 2013). Instead, grounded cognition theories assume that modal activations can occur in the absence of any vivid sensory or motor experience (Kemmerer, 2015; Kiefer, Sim, Herrnberger, Grothe, & Hoenig, 2008). In fact, activity in modal brain regions has also been observed for masked words, which were not consciously perceived (Trumpp, Traub, & Kiefer, 2013b; Trumpp, Traub, Pulvermüller, & Kiefer, 2014).

Another core assumption of recent grounded cognition theories is that conceptual representations are flexible (Kiefer & Pulvermüller, 2012; Kuhnke, Kiefer, & Hartwigsen, 2020) in the sense that the feature composition of concepts depends on the task or situation at hand (Barsalou, 1982). Pulvermüller (2018) recently provided an explanation for task-related flexibility within modal brain areas based on a neurobiologically inspired model. At a mechanistic level, task-related conceptual flexibility is seen to be a result of cortical gain control processes. Depending on the specific task, gain control of cortical activation is realized through feedback loops regulating excitation or inhibition processes within modality-specific brain areas, respectively. It is assumed that task-related modulations of cortical activity result from attention shifts toward or away from sensorimotor meaning aspects.

Recently developed hybrid theories combine assumptions made by amodal and grounded cognition theories by proposing an interaction between modality-specific, multimodal and amodal conceptual hub areas for conceptual processing (Kiefer & Harpaintner, 2020; Kiefer & Pulvermüller, 2012; Kuhnke et al., 2020; Patterson et al., 2007). These so-called hub and spokes models propose hub regions to store unifying non-physical semantic information while still being connected with concrete-experiential regions (Patterson & Ralph, 2016; Ralph, Jefferies, Patterson, & Rogers, 2017).

While the representation of concrete concepts can be accommodated by grounded cognition theories, including hybrid theories, in a quite straightforward manner, the mere existence of abstract concepts is often interpreted as proof of amodal theories (Mahon & Caramazza, 2008) since abstract concepts do not refer to a physical referent. In a similar vein, Paivio’s Dual Coding Theory (1986) claimed that concrete concepts are represented through both, a visual imagery and a verbal semantic system. Abstract concepts, in contrast, are assumed to be represented exclusively within the verbal semantic system.

In order to better account for the representation of abstract concepts, refined grounded cognition theories aimed to specify the relation between modality-specific brain systems and abstract concepts (see also Kiefer & Harpaintner, 2020; Pulvermüller & Henningsen, this issue). For instance, the Conceptual Metaphor Theory (Lakoff & Johnson, 1980) claims that the meaning of abstract concepts results from sensorimotor information originating from metaphoric relations to concrete concepts (see Desai, this issue). Barsalou and Wiemer-Hastings (2005) emphasize the importance of situational perceptions and therefore of direct sensorimotor experience in the constitution of the meaning of abstract concepts. They assume that conceptual knowledge is instantiated by partly simulating sensorimotor experiences made in specific situations during concept acquisition. The grounding of abstract concepts is thus thought to be based on simulations in modal brain systems. Considering “beauty,” for example, might simulate the visual scene of the admiration of the Starry Night described in the beginning of this article (see also Vergallito, Guenther, Marelli, & Petilli, this issue).

Based on the empirical findings that the semantic content of abstract concepts is highly heterogeneous (see below; Barca, Mazzuca, & Borghi, 2017; Harpaintner, Trumpp, & Kiefer, 2018; Kiefer & Harpaintner, 2020; Muraki, Sidhu, & Pexman, this issue; Wiemer-Hastings, Krug, & Xu, 2001; Wiemer-Hastings & Xu, 2005), grounded cognition approaches extended their theoretical framework by emphasizing the importance of additional types of conceptual information besides sensorimotor information. Linguistic/verbal (Barca et al., this issue; Language And Situated Simulation Theory, Barsalou, Santos, Simmons, & Wilson-Mendenhall, 2008; Words As Social Tools Approach, Borghi & Binkofski, 2014; Louwerse's Symbol Interdependency Hypothesis, Louwerse, 2011), social (Barsalou & Wiemer-Hastings, 2005; Borghi & Binkofski, 2014), affective (Affective Embodiment Account, Kousta, Vigliocco, Vinson, & Andrews, 2009) and introspective (Barca et al., this issue; Kiefer & Barsalou, 2013) experiential information is thought to be essential in the representation of abstract concepts.

Evidence favoring the grounded cognition framework mainly comes from studies on concrete concepts. Behavioral (e.g., Garcia & Ibanez, 2016), neuroimaging studies (e.g., Kiefer et al., 2008) as well as transcranial magnetic stimulation studies (TMS; e.g., Pulvermüller, Hauk, Nikulin, & Ilmoniemi, 2005a) or neuropsychological studies (e.g., Trumpp, Kliese, Hoenig, Haarmeier, & Kiefer, 2013a) demonstrated the involvement of modal brain systems in conceptual processing. Several electroencephalography (EEG) studies, which provide the time course of conceptual processing, found differential event-related potential (ERP) effects as a function of the modal information involved (e.g., Hauk & Pulvermüller, 2004; Trumpp et al., 2014; Martin, 2006 #83).

In line with the notion of conceptual flexibility, previous studies furthermore showed a modulation of cortical activity during conceptual processing depending on the requested task (Hoenig, Sim, Bochev, Herrnberger, & Kiefer, 2008; Popp, Trumpp, & Kiefer, 2019; van Dam, van Dijk, Bekkering, & Rueschemeyer, 2012). Deep conceptual decision tasks, in which retrieval of feature-specific information is necessary, led to modality-specific effects in several studies (Papeo, Vallesi, Isaja, & Rumiati, 2009; Popp et al., 2019; Sato, Mengarelli, Riggio, Gallese, & Buccino, 2008). In contrast, shallow lexical decision tasks, in which conceptual retrieval is not task-relevant but occurs through associative processes, led to a diminution or even a disappearance of differential effects (Papeo et al., 2009; Popp et al., 2019; Sato et al., 2008) illustrating that conceptual processing is highly flexible in the sense that it is dependent on the task at hand.

While the involvement of the sensorimotor system for the processing of concrete concepts is well documented, corresponding evidence with regard to abstract concepts is limited (for a review see Kiefer & Harpaintner, 2020). One line of evidence indicating a foundation of abstract concepts in perception and action comes from rating studies (Binder et al., 2016; Lynott & Connell, 2009, 2013; Troche, Crutch, & Reilly, 2014, 2017; van Dantzig, Cowell, Zeelenberg, & Pecher, 2011) and property generation studies (Barsalou & Wiemer-Hastings, 2005; Harpaintner et al., 2018) examining the subjective semantic content of abstract concepts. Besides verbal, emotional and introspective information, information associated with sensory and motor experiences was found to be related to all kind of concepts, regardless of their concreteness/abstractness level (Barsalou & Wiemer-Hastings, 2005). For instance, a property generation study (Harpaintner et al., 2018 321), which examined a large set of abstract concepts, yielded substantial proportions of generated verbal associations as well as social, emotional and introspective properties. However, sensorimotor properties were generated most frequently in response to abstract concepts in this study. Additional hierarchical cluster analyses indicated the existence of specific subgroups of abstract concepts characterized by the dominance of certain modal features, with one of those clusters showing a dominance of sensorimotor features. In terms of quantity, visual and motor properties played the most crucial role within the sensorimotor feature category. This indicates that abstract concepts are quite heterogeneous (see also Kiefer & Harpaintner, 2020; Muraki et al., this issue) with regard to their semantic content and cannot be contrasted as a uniform category with concrete concepts. Of course, rating and property generation studies do not indicate whether sensorimotor information related to abstract concepts is represented in corresponding modality-specific brain areas, as it is stated by grounded cognition theories (Kiefer & Pulvermüller, 2012). This information can only be provided by neuroimaging studies.

Several neuroimaging studies investigated the involvement of the modal cortex during the processing of abstract concepts (for a review see Kiefer & Harpaintner, 2020). In line with the suggested crucial role of mental states (Wilson-Mendenhall, Simmons, Martin, & Barsalou, 2013), social constellations (Wilson-Mendenhall et al., 2013) and emotions (Vigliocco et al., 2014) for the representation of abstract concepts, increased brain activity in corresponding neural networks has been found. Results of a few experiments furthermore suggest an association between sensorimotor cortex and abstract concepts: Processing of numerical concepts (e.g., "nine", see also Glenberg, Fischer, Shaki, & Doricchi, this issue; Tschentscher, Hauk, Fischer, & Pulvermüller, 2012), abstract concepts related to mental states (e.g., "thought", Dreyer & Pulvermüller, 2018) as well as abstract emotion words (Dreyer & Pulvermüller, 2018; Moseley, Carota, Hauk, Mohr, & Pulvermüller, 2012) was associated with enhanced activity in the motor cortex. A lesion study (Dreyer et al., 2015) furthermore suggested the motor cortex to be causally involved in the processing of abstract emotion words. Processing the single abstract concept “observe” led to increased activity in auditory and visual cortices (Wilson-Mendenhall, Barrett, Simmons, & Barsalou, 2012) and even highly abstract physical concepts were associated with enhanced activity in widespread modality-specific brain areas (Mason & Just, 2016). Finally, a recent fMRI study (Harpaintner, Sim, Trumpp, Ulrich, & Kiefer, 2020) indicated that abstract concepts with either a strong motor or a strong visual feature dominance were processed in corresponding modal cortices, which were also activated by action and perception. While the processing of motor-related abstract concepts, similarly as the execution of real movements, was associated with an enhanced BOLD signal in frontal and parietal motor regions, the processing of vision-related abstract concepts specifically elicited enhanced activation in temporo-occipital visual brain areas, similarly as the observation of object pictures (see also Vergallito et al., this issue). Although a shallow lexical decision task was used in this earlier study, which did not encourage semantic elaboration or imagery, it cannot be ruled out that sensorimotor activity was driven by these kinds of strategic processes. Furthermore and most importantly, it cannot be ruled out that sensorimotor activity occurred relatively late during task performance through spreading activation, after a putatively amodal concept had been accessed (Mahon, 2015a, 2015b). However, due to the poor temporal resolution of the fMRI, the time course of feature-specific processing of abstract concepts could not be determined in this earlier study.

Due to their excellent time resolution in the range of milliseconds, event-related potentials (ERPs) are the ideal tool to track the time course of brain activity elicited by conceptual processing. As already indicated above, several ERP studies investigated the processing of some subgroups of concrete concepts (Barber, Kousta, Otten, & Vigliocco, 2010; Grisoni, Dreyer, & Pulvermüller, 2016; Hauk & Pulvermüller, 2004; Kiefer, 2001, 2005; Kiefer et al., 2008; Martin, Hauk, & Pulvermüller, 2006; Popp, Trumpp, & Kiefer, 2016; Trumpp et al., 2013a, 2014). Concrete concepts with a dominance of specific feature types elicited differential ERP effects with a distinct topography suggesting that they are generated in different brain areas. For instance, concepts with a dominance of motor features such as “hammer” were associated with more positive ERPs over the central and frontal scalp, whereas more positive occipito-parietal ERPs were found for concepts with a dominance of visual features such as “cat” (Kiefer, 2001, 2005; Proverbio, Del Zotto, & Zani, 2007). Furthermore, differential ERPs as a function of feature type started at about 150 ms after target onset (Hauk & Pulvermüller, 2004; Hoenig et al., 2008; Kiefer, Sim, Helbig, & Graf, 2011; Proverbio et al., 2007; Pulvermüller, Härle, & Hummel, 2000). The rapid onset of these ERP effects indicates that they reflect early lexico-semantic processes and not later semantic elaboration, imagery or spreading activation processes as predicted by amodal approaches (Mahon & Caramazza, 2008).

To the best of our knowledge, electrophysiological investigations of abstract concepts are predominantly limited to the comparison of concrete vs. abstract concepts (for an exception see Bechtold, Bellebaum, Egan, Tettamanti, & Ghio, 2019). In contrast to abstract concepts, concrete concepts elicited more negative scalp potentials between 300 and 500 ms after target onset (Adorni & Proverbio, 2012; Barber, Otten, Kousta, & Vigliocco, 2013). This so-called N400 concreteness effect, which has been interpreted to reflect greater integration of multimodal information for concrete than abstract concepts (Barber et al., 2013), has been observed across a broad variety of tasks and stimuli (Bechtold, Ghio, & Bellebaum, 2018), even though differential ERPs of earlier (P1—N1; Wirth et al., 2008) and later (N700; West & Holcomb, 2000) latencies have also been found (Adorni & Proverbio, 2012). However, as already indicated above, contrasting abstract concepts as an undifferentiated conceptual category with concrete concepts  is questionable when taking into account the heterogeneity of abstract concepts. Also, note that the contrast abstract concepts vs. concrete concepts as a whole renders it difficult to compare the N400 concreteness effect with ERPs related to feature-specific effects described above, which are based on the comparison between different subgroups of concrete concepts (e.g., motor- vs. vision-related concrete concepts).

Abstractness does not only modulate the amplitude of particular ERP components, but also seems to affect latency of specific electrophysiological effects with later ERP effects for abstract concepts than for concrete concepts (Borghi et al., 2017): Palazova, Sommer and Schacht (2013) found a delayed emotion related early posterior negativity effect (EPN), an effect believed to reflect attention shifting to word meaning, for abstract than for concrete verbs of different valence in a lexical decision task. Bardolph and Coulson (2014) presented their participants words literally or metaphorically associated with vertical space (literal: ascend, descend; metaphorical: victory, poverty) while moving marbles either up- or downwards. They found early (200–300 ms after word onset) ERP congruency effects for literal words, while metaphorically related words elicited ERP congruency effects only 500 ms after word onset indicating that participants integrated abstract concepts and spatial schemas but, compared to concrete concepts, not in a rapid manner (Borghi et al., 2017). Some researchers considered the differential ERPs as an indication that abstract and concrete concepts are processed in different neural systems (Dove, 2011).

Furthermore, the later emergence of ERP effects in abstract than in concrete concepts has been interpreted to reflect mental imagery instead of lexico-semantic processes (Adorni & Proverbio, 2012; Barber et al., 2013; Bechtold et al., 2018; Borghi et al., 2017). As outlined above, it has been argued that late effects, presumably indexing post-conceptual imagery processes (Machery, 2007), do not preclude the existence of amodal conceptual representations, which are accessed earlier. For that reason, only demonstration of early sensorimotor activity during a conceptual task, reflecting access to conceptual representations rather than post-conceptual processes, can be taken as unequivocal evidence for grounded cognition theories (Kiefer & Pulvermüller, 2012). Returning to the ERPs just mentioned, however, results are heterogeneous (Adorni & Proverbio, 2012) with some studies indicating early ERP effects in the time window of P1–N1 for abstract concepts, speaking against late mental imagery processes (Wirth et al., 2008). Additionally, previous ERP studies simply contrasted abstract with concrete concepts and did not differentiate between possible conceptual subgroups of abstract concepts. As outlined above, rating (Binder et al., 2016; Lynott & Connell, 2009, 2013; Troche et al., 2014, 2017; van Dantzig et al., 2011) and property generation (Barsalou & Wiemer-Hastings, 2005; Harpaintner et al., 2018) studies suggest subgroups of abstract concepts with a differential conceptual feature composition. In line with this reasoning, our recent fMRI study showed that abstract concepts with an empirically defined dominance of visual vs. motor features activated corresponding modal cortex (Harpaintner et al., 2020).

In the present ERP study, we therefore systematically investigated the time course of abstract noun processing. We adopted the theory-driven approach and the stimuli from our previous fMRI study (Harpaintner et al., 2020) and compared electrophysiological responses to specific subgroups of abstract concepts with a known feature composition. Stimuli were motor- and vision-related abstract concepts as determined by a previous property listing study (Harpaintner et al., 2018), in which motor and visual features were generated the most. Based on this property listing study, 32 abstract words highly related to motor properties (e.g., “fitness”) and 32 abstract words highly related to visual properties (e.g., “similarity”) were selected. Please note, that for better readability, these two word lists are called motor and visual abstract concepts from now on, respectively.

This study aimed to address three specific research questions: Firstly, we asked whether feature-specific ERP effects for motor and visual abstract concepts would be similarly observed as for concrete concepts. Secondly, as amodal theories attribute sensorimotor activation to later post-conceptual imagery, elaborative or spreading activation processes, we assessed whether possible differential ERP effects would emerge in early (between 150 and 300 ms) or in later time windows. Visual word recognition is completed at about 150 ms after word onset (Pulvermüller, Shtyrov, & Ilmoniemi, 2005b), and full access to a concept is assumed to be mandatory for imagery (Kosslyn, 1994). For that reason, ERP effects observed immediately after 150 ms most likely reflect semantic access and not imagery. Thirdly, we tested whether processing of abstract concepts is prone to conceptual flexibility and assessed whether a deep conceptual task leads to earlier feature-specific ERP effects compared to a shallow conceptual task, similar to observations in concrete concepts. In a deep conceptual task, retrieval of conceptual information is mandatory for task performance, for instance, when the semantic relatedness of two words has to be determined (Simmons, Hamann, Harenski, Hu, & Barsalou, 2008). In contrast, in a shallow conceptual task, retrieval of conceptual knowledge is not necessary for task performance, but occurs through associative links. For instance, visual word recognition in a lexical decision task (word/pseudoword decision) primarily depends on retrieval of lexical information, whereas access to conceptual information is assumed to occur auxiliary (Dilkina, McClelland, & Plaut, 2010).

In the first experiment, motor and visual abstract concepts, besides pseudowords, were presented within a shallow lexical decision task with a go/no-go response mode, in which retrieval of conceptual knowledge is not necessary for task performance, but occurs through associative links (Simmons et al., 2008). Furthermore, since the go/no-go response mode did not require an overt motor response in case of the critical word stimuli, interference with conceptual processing in the motor system was avoided (Schomers & Pulvermüller, 2016). In the second experiment, participants had to perform a deep conceptual decision task, in which the semantic relation between a context word and subsequent motor and visual abstract concepts had to be determined. Note that the tasks as realized in Experiments 1 and 2 do not only differ with regard to the mandatory requirement of semantic retrieval, but also with regard to the response mode (go/no-go response mode vs. two alternative forced choice) and relational processing (single word vs. relational word processing). Nevertheless, all these factors converge on the fact that deeper semantic processing is required in the conceptual decision task of Experiment 2 compared to the lexical decision task of Experiment 1. In both experiments, we expected different scalp potentials in response to motor and visual abstract concepts. Similar to previous observations on concrete concepts, motor abstract concepts should elicit more positive ERPs over the fronto-central scalp, whereas visual abstract concepts should be associated with more positive ERPs over the occipito-temporal scalp. Furthermore, the onset of feature-specific ERP effects should be modulated by task with earlier feature-specific ERP effects in the deep conceptual decision task as compared to the shallow lexical decision task, because this type of task demands retrieval of conceptual information (Papeo et al., 2009; Popp et al., 2019; Sato et al., 2008). In line with the grounded cognition framework, early ERP effects within 150–300 ms after target onset in response to motor and visual abstract concepts would suggest that feature-specific brain activity reflects rapid access to sensorimotor features and not later post-conceptual processes.

## Experiment 1

### Methods

#### Participants

Twenty-nine healthy, right-handed (according to Oldfield, 1971) native German-speaking students from Ulm University participated in the study. Six participants were excluded from the analysis due to excessive artifacts in the EEG recording. Final analysis included electrophysiological data of 23 participants (*M*_*age*_ = 22.7 years, range = 18–27 years, 12 females). Participants had normal or corrected-to-normal vision, were free from a history of neurological or psychiatric disorders and did not participate in a previous study of our laboratory using the same stimuli/procedure. Participants gave written informed consent and were paid 24 Euros or course credits for participation. Procedures for Experiments 1 and 2 were approved by the Ethical Committee of Ulm University and adhere to the tenets of the Declaration of Helsinki.

#### Stimuli

Sixty-four abstract words (see Online Resource 1 for the full set of verbal stimuli) served as critical stimuli in the lexical decision task with a go/no-go response mode. Additional 32 pseudowords (go trials) served as distractors and were not further analyzed. The same verbal stimuli have been used and described in detail in a previous study (Harpaintner et al., 2020). Critical abstract words were embedded as no-go trials and were thus presented without interference from a motor response. Half of those abstract words (32 words) had a strong link to motor properties, whereas the other half had a strong link to visual conceptual properties, as determined on the basis of a previous study (Harpaintner et al., 2018). In this study, participants (not participating in the present study) had to generate properties for 296 abstract concepts, which were subsequently categorized according to modality-specific and verbal contents (for further details see Harpaintner et al., 2018). At least 15% of properties that have been coded as motor or visual had to be generated with regard to the respective abstract concept in order to be assigned to the motor or visual abstract subcategory, respectively. Note that a proportion of 15% modality-specific properties exceeded the mean proportions of motor (*M* = 13.1%) and visual (*M* = 14.8%) properties generated for all 296 concepts in our previous study. Furthermore, the difference of generated motor and visual properties of each word had to be at least 10% in order to achieve a substantial difference in the conceptual feature dominance of the two subcategories.

The chosen motor (*M*_motor_ = 31.60%, *M*_visual_ = 8.97%; e.g., “fitness”) and visual (*M*_visual_ = 30.18%, *M*_motor_ = 6.49%; e.g., “beauty”) abstract concepts were carefully matched with regard to possible confounding conceptual features (proportion of generated acoustic features, valence, arousal, concreteness/abstractness, familiarity) and psycholinguistic variables (word length, lemma frequency, bigram and trigram frequency; Table 1; for further details see Harpaintner et al., 2020; Harpaintner et al., 2018). Additionally, a previous pilot study (Harpaintner et al., 2020), which used a classical lexical decision task and thus made behavioral data available for pseudowords as well as for motor and visual abstract concepts, confirmed comparable task difficulty for motor and visual abstract concepts, as measured by reaction times (*M*_motor_ = 578.60 ms, *M*_visual_ = 566.91 ms, *p* = 0.78) and error rates (*M*_motor_ = 0.50%, *M*_visual_ = 0.42%, *p* = 1.00). Note that we did not match the number of derived words across the two conditions (*n*_motor_ = 25 vs. *n*_visual_ = 18). However, when tested with help of a *χ*^*2*^-test, there was no significant difference in the distribution of derived words between motor and visual abstract words (*χ*^*2*^(1) = 3.47, *p* = 0.062). Possible confounding influences related to differential numbers of derived words between the conditions on ERPs were therefore unlikely.

**Table 1:** Mean values and standard deviation (in parenthesis) of conceptual and psycholinguistic variables for motor and visual abstract concepts

	Motor abstract concepts	Visual abstract concepts	Motor vs. visual (*p*-values)^a^
Proportion motor properties	0.32 (0.12)	0.06 (0.04)	*p* < .001
Proportion visual properties	0.09 (0.06)	0.30 (0.09)	*p* < .001
Proportion acoustic properties	0.03 (0.04)	0.04 (0.05)	*p* = .536
Concreteness/abstractness^b^	2.65 (0.61)	2.49 (0.53)	*p* = .276
Familiarity^b^	4.18 (0.67)	4.07 (0.57)	*p* = .454
Valence^b^	0.51 (1.83)	0.26 (1.87)	*p* = .582
Arousal^b^	2.74 (0.78)	2.62 (0.95)	*p* = .561
Word length	8.19 (2.58)	7.88 (2.06)	*p* = .595
Lemma frequency p. Mio	63.14 (91.72)	38.21 (45.17)	*p* = .173
Character bigram frequency p. Mio	652,936.87 (379,468.24)	631,388.19 (278,144.87)	*p* = .796
Character trigram frequency p. Mio	229,175.80 (124,964.62)	218,018.45 (103,100.90)	*p* = .698

^a^Depicted *p*-values were obtained using two-tailed *t*-tests

^b^Scales of the items: concreteness/abstractness: six-point Likert scale with the poles “abstract” (1) and “concrete” (6); familiarity: six-point Likert scale with the poles “low familiarity” (1) and “high familiarity” (6); valence: six-point Likert scale with the poles “negative” (-3) and “positive” (+ 3); arousal: self-assessment manikins (Bradley & Lang, 1994) with the poles “weak” (1) and “strong” (5)

Thirty-two pseudowords were created by replacing one consonant and one vowel of abstract concepts not used in the experimental conditions by another consonant and vowel. Pseudowords thus consisted of meaningless but pronounceable letter strings (e.g., “Antordirung”). Pseudowords and words of the experimental conditions did not differ with regard to their word length (*M*_motor_ = 8.19, *M*_visual_ = 7.88, *M*_pseudo_ = 7.94, *F*(2,93) = 0.168, *p* = 0.85).

#### Procedure

Participants were seated in front of a CRT computer screen synchronous with the screen refresh (refresh rate: 16 ms) at a distance of 70 cm resulting in a viewing angle subtending about 3° horizontally and 1° vertically. Words were presented in a randomized order as white letters (font size: 16 point character height) on a black background in the middle of the screen. Each trial started with a fixation cross of 500 ms duration followed by the target lasting for 400 ms. Participants had to decide whether the presented stimulus is a real German word or a pseudoword. A pseudoword indicated a go trial, and participants were instructed to press a button on the response keyboard with the index finger of their right hand. If the stimulus was a real German word, participants should passively read the word, but were instructed not to react (no-go trial). The assignment of abstract words to the no-go condition prevented an overt motor activity to the critical word stimuli. Participants were instructed to decide as fast and accurately as possible. The screen remained blank until a response was given or for 1400 ms in case of a no-go trial or a missed response, respectively. At the end of each trial, three hash marks lasting for 2000 ms indicated a pause between the trials. Stimulus presentation and behavioral data acquisition were controlled by the Experimental Runtime System software package (Berisoft, Frankfurt, Germany). A training with 20 stimuli not used in the main experiment preceded the experimental session.

#### EEG-recording, signal extraction, data analysis

The study was carried out in a sound attenuated, dimly illuminated and electrically shielded cabin. Participants were comfortably seated in an upright position and were instructed via detailed written and verbal instructions. To ensure complete understanding of the instructions, participants had to practice the task in a training session preceding the main experiment as indicated above. Participants were furthermore encouraged to blink only during the breaks and to stay relaxed during the whole EEG recordings in order to avoid ocular and movement artifacts.

Scalp voltages were continuously recorded at a sampling rate of 500 Hz (low-pass filter: 70 Hz, 24 dB/octave attenuation, 50 Hz notch filter) by BrainAmp amplifiers and BrainVision Recorder software (BrainProducts, Gilching, Germany) using 64 equidistant Ag/AgCl electrodes mounted in an elastic textile cap (EasyCap, Herrsching, Germany) with a cap size determined by the subjects' head circumference (52, 54, 56, 58, or 60 cm). An electrode between FCz and Cz was used as recording reference; the ground electrode was positioned between AFz and Fz. Eye movements were monitored with supra- and infra-orbital electrodes and with electrodes on the external canthi. All EEG electrode impedances were maintained below 5 kΩ.

EEG data were processed offline by BrainVision Analyzer 2.0 (BrainProducts, Gilching, Germany). After digitally filtering (high pass: 0.1 Hz, 12 dB/octave, low pass: 30 Hz, 24 dB/octave, 50 Hz notch filter) the EEG data, Independent Component Analysis (ICA) was used to remove ocular artifacts (Makeig, Jung, Bell, Ghahremani, & Sejnowski, 1997). Hjorth nearest neighbors interpolation replaced data of single noisy electrodes by interpolated data of four surrounding electrodes. Continuous EEG data were segmented starting 150 ms prior to target presentation, which served for baseline correction, and ended 1000 ms after target onset. Segments exhibiting amplitudes of more than 70 µV or less than -70 µV, showing voltage steps greater than 50 µV/ms and exhibiting 120 µV differences of values in intervals, were automatically excluded as artifacts from analyses. The remaining artifact-free EEG segments of trials with correct responses were averaged synchronous to the onset of the target separately for each experimental condition in each participant in order to extract individual ERPs. Thereafter, these ERPs were re-referenced to the average reference (Bertrand, Perrin, & Pernier, 1985).

To test for significant differences between conditions across all electrode sites and the whole ERP time window, statistical analyses were performed using BESA Statistics 2.0 (BESA GmbH, Graefelfing, Germany). To avoid the problem of multiple comparisons due to a large number of time points and channels, BESA statistics makes use of a combination of permutation testing and data clustering (Ernst, 2004; Maris & Oostenveld, 2007). In order to exclude post-lexical semantic processes, analyses focused on effects prior to 800 ms after target onset. The initial statistics used for the subsequent permutations were based on two-tailed paired samples *t*-tests comparing ERP data in response to motor vs. visual abstract concepts. A cluster value consisting of the sum of all *t*-values derived from a random permutation procedure (1000 permutations) was determined for each cluster such that the significance of the initial clusters could be determined based on the distribution of the calculated cluster values after permutation. Level of significance was defined as *p* < 0.05, corrected for multiple comparisons. The mean number of artifact-free EEG segments of trials with correct responses was 31.51 (*SD* = 0.66) for motor and 31.56 (*SD* = 0.92) for visual abstract concepts. A two-tailed paired samples *t*-test confirmed that the number of segments did not significantly differ between conditions (*t*(22) = -0.24, *p* = 0.81).

Because of the go/no-go design of the lexical decision task, in which participants reacted only to the theoretically irrelevant pseudowords, analysis of behavioral data in form of reaction times (RTs) were not informative. However, mean error rates (ERs) were calculated for each participant and each condition (motor, visual, pseudo). Subsequent univariate repeated measures analyses of variance (ANOVA) were carried out in order to investigate whether error rates differed significantly between the conditions. Level of significance was defined as *p* < 0.05.

## Results

### Behavioral data

Analysis of behavioral data yielded a mean ER of 1.27% (*SD* = 1.44%) showing that participants performed the task carefully. Participants failed to respond to pseudowords in 1.22% of the trials (*SD* = 2.05%). ER in word trials (failure to withhold response) was 1.36% for motor abstract concepts (*SD* = 1.84%) and 1.22% for visual abstract concepts (*SD* = 2.79%). A univariate repeated measures ANOVA revealed no significant differences in mean ERs between motor and visual abstract concepts and pseudowords, respectively (*F*(2,44) = 0.03, *p* = 0.97).

### Electrophysiological data

**Fig. 1:**
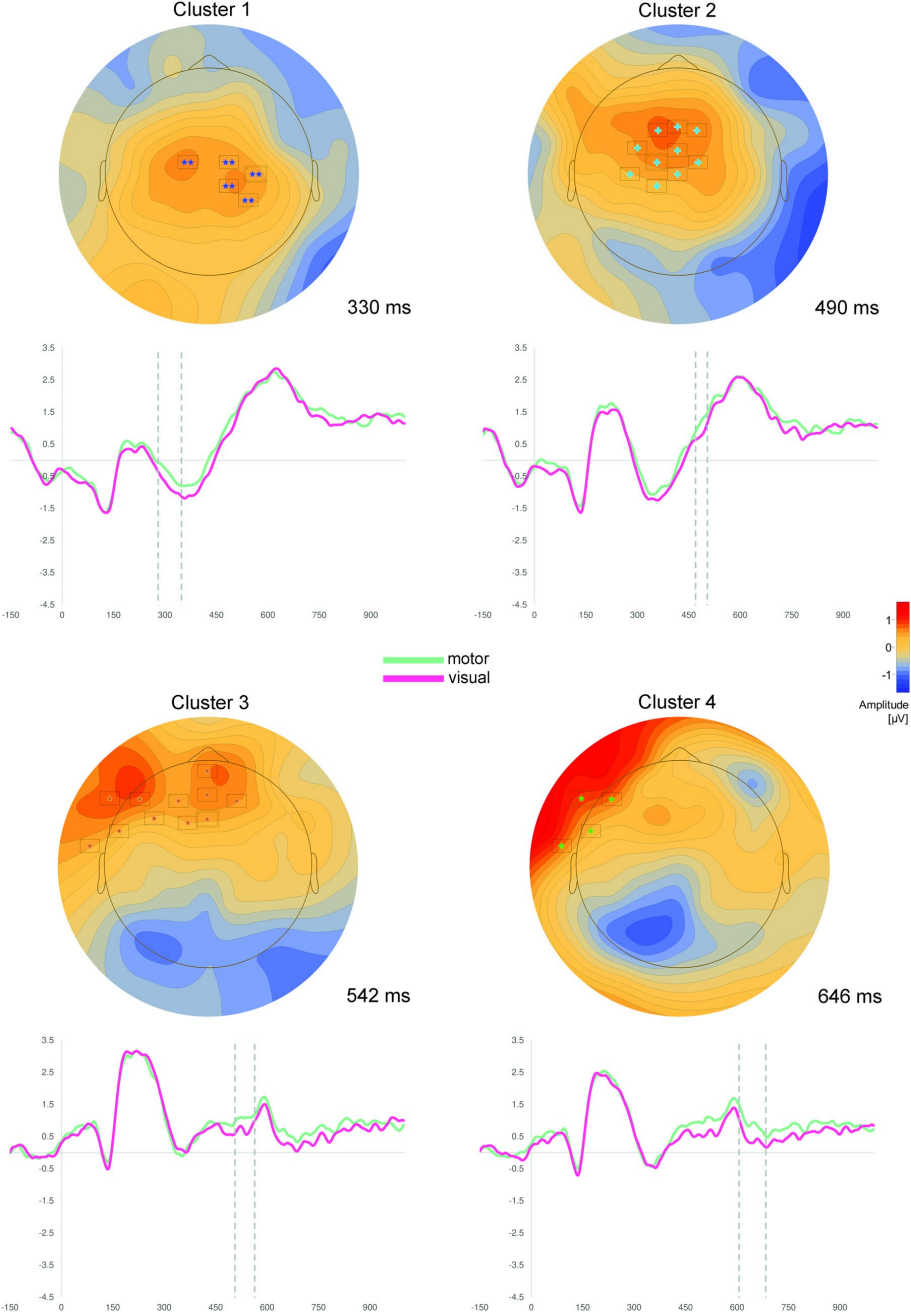
Results of cluster permutation tests of Experiment 1. Depicted are clusters, which show significantly more positive scalp potentials for motor compared to visual abstract concepts. Above: Topographic map of each cluster at the time point of the highest *t*-value across all electrodes. Only electrodes with significant *t*-values (*p* < .05) at the specific time point are depicted (for all electrodes see Table 2). Below: ERPs averaged over all electrodes of the respective cluster. Dotted lines indicate the significant time window of the cluster

**Fig. 2:**
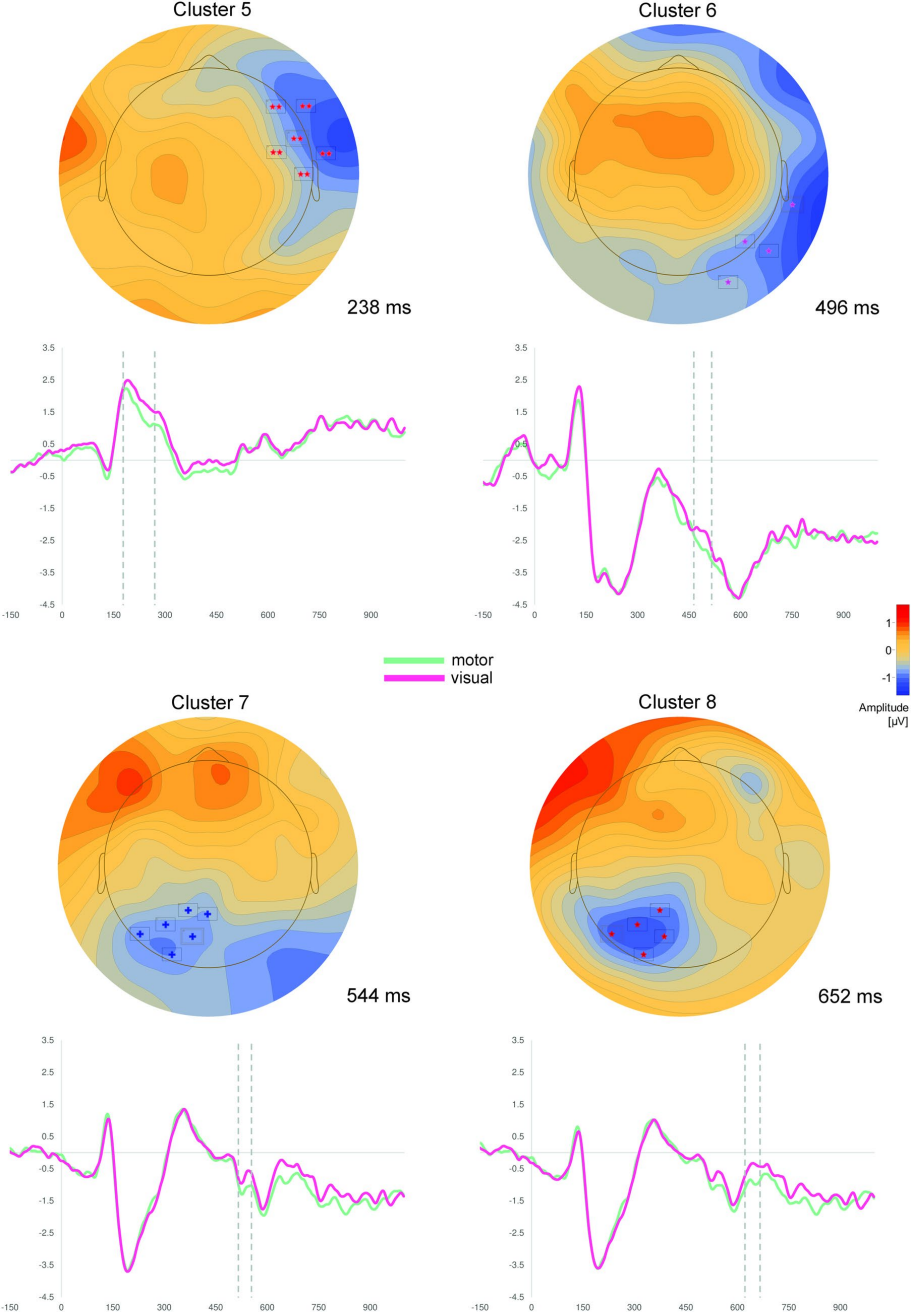
Results of cluster permutation tests of Experiment 1. Depicted are clusters, which show significantly more positive scalp potentials for visual compared to motor abstract concepts. Above: Topographic map of each cluster at the time point of the highest *t*-value across all electrodes. Only electrodes with significant *t*-values (*p* < .05) at the specific time point are depicted (for all electrodes see Table 2). Below: ERPs averaged over all electrodes of the respective cluster. Dotted lines indicate the significant time window of the cluster

Cluster permutation tests revealed significant differences between the processing of motor vs. visual abstract concepts in eight clusters (Table 2, Figs. 1, 2).

**Table 2:** Results of cluster permutation tests of Experiment 1

Polarity	Cluster^a^	Electrodes within cluster	Time window (ms)	*p*-value
Motor > visual	1	FCz, FC1, CP1, Cz, CP4, C4, FC2, CP2	280–348	< .005
	2	C3, FC3, F1, Fz, FCz, FC1, CP1, Cz, F2, FC2	470–504	< .05
	3	F9, FT9, FT7, AF7, FPz, AFz, AF3, F5, FC5, F1, Fz, AF4	506–564	< .05
	4	F9, FT9, FT7, AF7, FC5, FC3, F1, FCz	606–684	< .05
Visual > motor	5	F10, FT10, T8, FT8, AF8, FP2, AF4, F6, FC6	178–270	< .005
	6	TP10, P10, O10, Iz, O2, P8, TP8	464–516	< .05
	7	O1, P7, PO3, PO1, P1, Oz, Pz	516–554	< .05
	8	O1, P7, TP7, PO3, PO1, P1, Pz	622–666	< .05

^a^Reported clusters were sorted by polarity and ordered by time window (early → late)

Four fronto-centrally located clusters were characterized by more positive scalp potentials in response to motor vs. visual abstract concepts (Fig. 1): Clusters 1 and 2 comprised overlapping fronto-central electrodes in the time windows from 280 to 348 ms and from 470 to 504 ms. Cluster 3 and 4 were more lateralized to the left hemisphere and encompassed mostly frontal electrodes in the time windows 506 to 564 ms and 606 to 684 ms, respectively.

Similarly, four clusters were characterized by more positive scalp potentials in response to visual vs. motor abstract concepts (Fig. 2): The earliest cluster, Cluster 5, showed significant differences in right fronto-temporal electrodes between 178 and 270 ms. The three remaining clusters were located more occipitally, with Cluster 7 (516–554 ms) and 8 (622–666 ms) being distributed over the left hemisphere and Cluster 6 (464–516 ms) being distributed over the right hemisphere, respectively.

## Discussion

Experiment 1 showed differential feature-specific ERP effects in response to motor and visual abstract concepts in a shallow lexical decision task. Motor abstract concepts were related to significantly more positive potentials over frontal and central scalp regions, whereas the processing of visual abstract concepts was specifically associated with more positive scalp potentials over parieto-occipital as well as over right fronto-temporal scalp regions. ERP-effects began to emerge at 178 ms after target presentation. However, later effects beyond 300 ms were also observed.

In spite of the shallow nature of the lexical decision task, we found early feature-specific ERP effects in the present study. This contrasts studies which suggest a diminution or a disappearance of differential effects when using shallow tasks (Papeo et al., 2009; Popp et al., 2019; Sato et al., 2008). The early emergence of feature-specific scalp potentials indicates that effects reflect rapid access of motor and visual information. At the same time, post-conceptual processes such as imagery, semantic elaboration or spreading activation might also take place after this initial conceptual access, as indicated by the relatively late differential ERPs.

The time course, the polarity as well as the topography of the differential ERP effects parallel results from earlier studies, which examined the processing of concrete concepts (Kiefer, 2001, 2005; Sim & Kiefer, 2005; Trumpp et al., 2014). These findings indicated that the processing of concrete motor concepts is associated with more positive potentials in fronto-central scalp regions (Hauk & Pulvermüller, 2004; Kiefer, 2001, 2005; Popp et al., 2016; Pulvermüller, Lutzenberger, & Preissl, 1999; Pulvermüller, Preissl, Lutzenberger, & Birbaumer, 1996; Trumpp et al., 2014), whereas the processing of concrete visual concepts was related to significantly more positive ERPs in temporal and occipital scalp regions (Kiefer, 2001, 2005; Martin et al., 2006; Pulvermüller et al., 1999, 1996; Sim & Kiefer, 2005). However, in contrast to earlier findings within the concrete concept class, we found feature-specific ERPs in response to visual abstract concepts also in right fronto-temporal electrodes of Cluster 5. This difference might be due to differential conceptual representations of the two concept classes (concrete vs. abstract) or might reflect interindividual differences in neuroanatomy of different samples. Moreover, although care must be taken with regard to EEG and spatial localization, the source of the fronto-temporal electrode cluster may be located in the anterior part of the fusiform gyrus, as it already has been indicated by a previous fMRI study (Harpaintner et al., 2020). Furthermore, it cannot be ruled out that the source of the ERPs is located in the temporal pole, a prominent candidate for a hub region (Patterson et al., 2007), although the previous fMRI study using the same stimulus material did not observe differential activity in this region. Note that we did not perform source analyses because of the low signal-to-noise-ratio in the context of the relatively small ERPs in the present study and because our primary focus was the neural time course of abstract concept processing. Focusing that, our results suggest that differential ERP effects in response to motor and visual abstract concepts emerge 178 ms after target presentation indicating rapid access of modal features during conceptual processing. However, later feature-specific effects beyond 300 ms might reflect post-conceptual processes as already outlined above. At this point, we want to highlight the fact that the clusters showing the most significant differences between the processing of motor and visual abstract concepts are the clusters, in which differential effects emerge prior to 300 ms after target onset, further supporting the idea that the results reflect early semantic access to motor and visual information.

## Experiment 2

In contrast to the first experiment, participants of Experiment 2 were asked to perform a deep conceptual decision task, in which the semantic relation between a context word and subsequent motor and visual abstract concepts had to be determined. We expected that feature-specific processing would be boosted during a deep conceptual decision task, which demands retrieval of conceptual information. This task-dependent modulation should be evident by earlier feature-specific ERPs in the deep task as compared to the shallow lexical decision task (Papeo et al., 2009; Popp et al., 2019; Sato et al., 2008).

### Methods

#### Participants

Thirty-three native German-speaking undergraduate students from Ulm University participated in Experiment 2. Participants were healthy, right-handed (Oldfield, 1971) and had normal or corrected-to-normal vision. None of them reported a history of neurological or psychiatric disorders. Again, participants did not take part in a previous study of our laboratory using the same stimuli/procedure. Due to excessive artifacts in the EEG recordings, five participants were excluded from the EEG analysis. Final EEG analysis thus included data of 28 (*M*_*age*_ = 23.4 years, range = 19–29 years, 14 females) participants. Analyses of behavioral data included only 27 participants, because the data of one participant got lost due to a technical error. Participants gave written informed consent and were paid 17 Euros or course credits for participation.

#### Stimuli

The same 32 motor and 32 visual abstract concepts of Experiment 1 were used as stimuli for Experiment 2. Additional 64 abstract concepts (see Online Resource 2 for the full set of verbal stimuli) of our previous property listing study (Harpaintner et al., 2018) characterized by a low portion of generated motor and visual properties (e.g., “thirst”; *M*_*motor/visual*_ < 12%) served as filler words (not further analyzed). For each motor and visual abstract concept, we selected a semantically related concrete context noun (matching condition; e.g., “bride–beauty”), and for every filler word we determined a semantically unrelated context noun (non-matching condition; e.g., “candle–thirst”), respectively. Thus, all critical motor and visual abstract words were presented in the contextual matching condition. Context words were chosen out of four concrete word categories: action (e.g., “sailing”), location (e.g., “store”), object (e.g., “candle”) and person (e.g., “bride”). Context words were matched across conditions with regard to the respective word category and their word length. Furthermore, word length of motor and visual abstract words as well as the filler words was matched.

In order to test whether relatedness between context and abstract words differs significantly between visual and motor words, we quantified relatedness by performing a Latent Semantic Analysis (LSA; for further details see Günther, Dudschig, & Kaup, 2015) with the help of the R package “LSAfun” (Günther & Günther, 2018). The LSA uses linguistic co-occurrences based on large corpora and assesses the degree of the occurrence of words in similar contexts (Landauer & Dumais, 1997). Note that we were not able to obtain cosines based on the used corpora (“dewak.100 k.lsa.rda”)^1^ for five/three of the word pairs in the motor/visual condition, respectively. Comparing the cosines of the remaining word pairs with help of Welch’s two sample *t*-test yielded no significant differences between the motor (*M* = 0.32, *SD* = 0.20) and visual (*M* = 0.38, *SD* = 0.16) conditions (*t*(50.43) = -1.11, *p* = 0.27) indicating that the two critical lists of word pairs were comparable with regard to relatedness. Possible confounding influences related to differential degrees of relatedness between the conditions on ERPs were therefore unlikely.

A pilot study with ten participants (not participating in the present study) using the same conceptual decision task as the main experiment (see procedure) and the subsequent univariate repeated measures ANOVA (motor vs. visual vs. filler) yielded significant differences in mean RTs between the conditions (*F*(2, 18) = 6.28, *p* < 0.05). According to a *post hoc* test (Bonferroni test), this difference was due to slower reaction times in response to filler words (*M* = 740.50 ms) as compared to visual abstract concepts (*M* = 693.75 ms, *p* < 0.05). Importantly, mean RTs in response to motor (*M* = 713.42 ms) and visual abstract concepts did not differ significantly (*p* = 0.47). A further ANOVA yielded no significant differences with regard to mean ERs between the conditions (*F*(2, 18) = 1.70, *p* = 0.21). Results thus confirmed that the critical conditions (motor vs. visual abstract concepts) were comparable with regard to their difficulty as measured by RTs and ERs. Note that the absence of significant differences in mean RTs and ERs further supports the claim that relatedness of the context words and the critical abstract words did not differ between the conditions.

#### Procedure

Experiment 2 was designed as a deep conceptual decision task, in which 64 critical motor and visual abstract words as well as 64 abstract filler words paired with 64 matching and 64 non-matching context nouns, respectively, were presented to the participants. The total number of 128 trials was randomly presented in four blocks of 32 trials each, separated by breaks. Words were presented as white letters (font size: 16 point character height) on a black background in the middle of the screen (viewing angle about 3° horizontally and 1°vertically). Each trial started with a fixation cross of 500 ms duration followed by the context noun lasting for 400 ms. After a clear screen of 500 ms, the abstract target word was presented for 400 ms. Participants had to decide as fast and accurately as possible whether the two words were semantically related or not, and were instructed to press a key with their right index finger in case of related word pairs and another key with their right middle finger in case of unrelated word pairs, respectively. The assignment of the conditions to reactions with the right index or middle finger was counterbalanced. The screen remained blank until a response was given. After another clear screen lasting for 500 ms, three hash marks lasting for 500 ms indicated a pause between the trials. A training with 12 word pairs not used in the main experiment preceded the experimental session.

#### EEG recording, signal extraction, data analysis

EEG data regarding the two critical conditions (motor and visual abstract concepts) were recorded and analyzed similarly as described in Experiment 1. Again, the number of artifact-free EEG segments did not differ significantly between motor (*M* = 28.07, *SD* = 2.33) and visual (*M* = 28.96, *SD* = 1.77) abstract concepts (*t*(27) = 1.60, *p* = 0.12).

Behavioral data, RTs and ERs, were analyzed using Statistica 13.1 (StatSoft, Tulsa, USA). For RT analysis, only trials with correct conceptual decisions were included. Outlier trials (± 2 *SD*) were excluded from the RT analysis. Individual mean RTs and ERs in response to motor, visual and filler abstract words were compared using repeated measures of analyses of variance (ANOVA). Level of significance was defined as *p* < 0.05.

## Results

### Behavioral data

Mean ER of the deep conceptual decision task was 5.79% (*SD* = 2.56%), which shows that participants performed the task carefully. A univariate repeated measures ANOVA revealed significant differences in mean ER between motor and visual abstract concepts and filler words, respectively (*F*(2, 52) = 8.97, *p* < 0.001). According to a *post hoc* test (Bonferroni test), this difference was due to lower ERs in response to filler words (*M* = 3.41%, *SD* = 2.91%) as compared to motor (*M* = 9.26%, *SD* = 6.85, *p* < 0.001) and visual (*M* = 7.06, *SD* = 4.71, *p* < 0.05) abstract concepts. Importantly, ERs did not differ significantly between motor and visual abstract concepts (*p* = 0.36).

A further univariate repeated measures ANOVA revealed a similar pattern with regard to mean RTs. Significant differences between the conditions were found (*F*(2, 52) = 16.79, *p* < 0.001), but again, a *post hoc* test (Bonferroni test) revealed that differences were due to slower RTs in response to filler words (*M* = 884.89 ms, *SD* = 208.46 ms) as compared to motor (*M* = 827.27 ms, *SD* = 151.99 ms, *p* < 0.001) and visual (*M* = 805.48 ms, *SD* = 149.23 ms, *p* < 0.001) abstract concepts. Mean RTs in response to motor vs. visual abstract concepts did not differ significantly (*p* = 0.39).

### Electrophysiological data

**Fig. 3:**
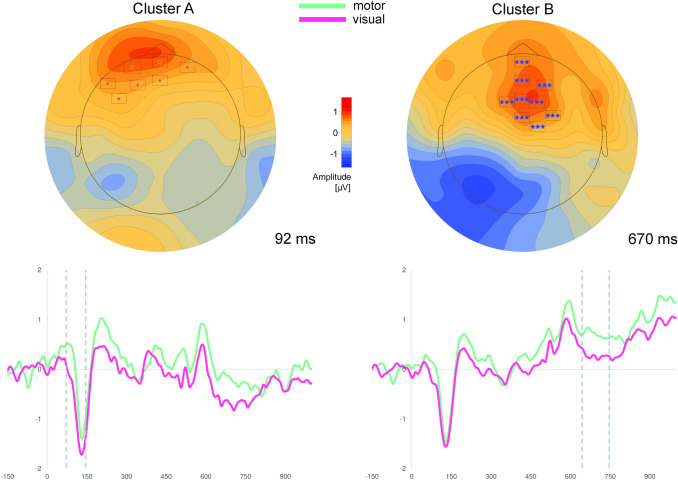
Results of cluster permutation tests of Experiment 2. Depicted are clusters, which show significantly more positive scalp potentials for motor compared to visual abstract concepts. Above: Topographic map of each cluster at the time point of the highest *t*-value across all electrodes. Only electrodes with significant *t*-values (*p* < .05) at the specific time point are depicted (for all electrodes see Table 3). Below: ERPs averaged over all electrodes of the respective cluster. Dotted lines indicate the significant time window of the cluster

**Fig. 4:**
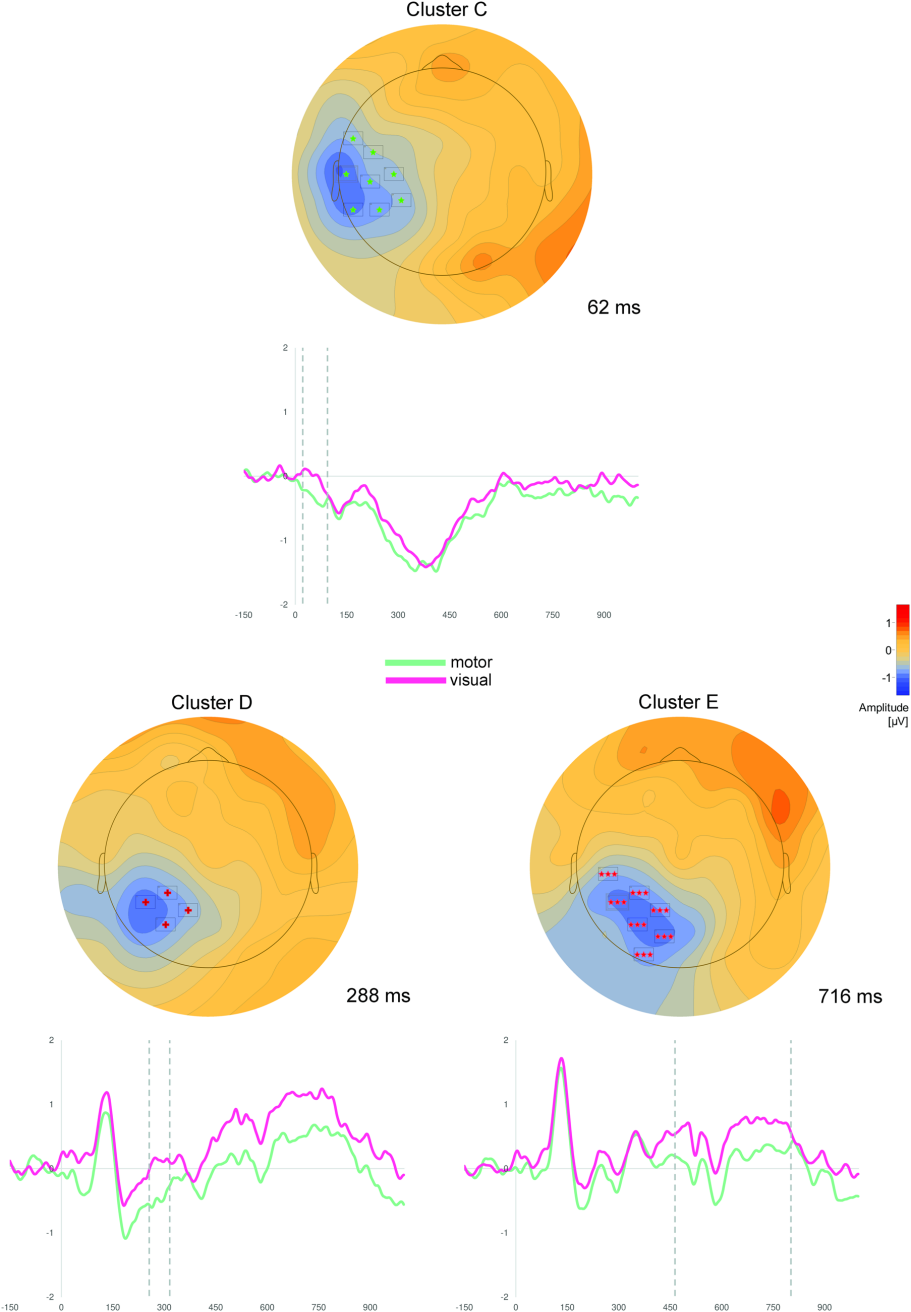
Results of cluster permutation tests of Experiment 2. Depicted are clusters, which show significantly more positive scalp potentials for visual compared to motor abstract concepts. Above: Topographic map of each cluster at the time point of the highest *t*-value across all electrodes. Only electrodes with significant *t*-values (*p* < .05) at the specific time point are depicted (for all electrodes see Table 3). Below: ERPs averaged over all electrodes of the respective cluster. Dotted lines indicate the significant time window of the cluster

Cluster permutation tests revealed significant differences between the processing of motor vs. visual abstract concepts in five clusters (Table 3, Figs. 3, 4).

**Table 3:** Results of cluster permutation tests for Experiment 2

Polarity	Cluster^a^	Electrodes within cluster	Time window	*p*-value
Motor > visual	A	AF7, FP1, FPz, AFz, AF3, F5, FP2, AF4, Nz	72–146 ms	< .01
	B	FP1, FPz, AFz, AF3, F1, Fz, FCz, F10, T8, FT8, AF8, FP2, AF4, F6, FC6, FC4, F2, FC2, Nz	644–746 ms	< .005
Visual > motor	C	TP9, P7, TP7, T7, FT7, FC5, C5, P5, PO3, CP3, C3, FC3, FCz, FC1, CP1, Cz, FC2, CP2	22–94 ms	< .005
	D	P7, TP7, C5, P5, PO3, P1, CP3	256–316 ms	< .05
	E	TP9, P9, O9, O1, P7, TP7, C5, P5, PO3, PO1, P1, CP3, CP1, Iz, Oz, O2, PO4, PO2, Pz, P2, CPz	464–802 ms	< .005

^a^Reported clusters were sorted by polarity and ordered by time window (early → late)

Cluster A, which encompassed mostly frontal electrodes in the time window between 72 and 146 ms, and Cluster B, which was fronto-centrally located in the time window from 644 to 746 ms, were characterized by more positive scalp potentials in response to motor vs. visual abstract concepts.

The reversed polarity effect (visual > motor) was found in three clusters located more posteriorly and more lateralized to the left hemisphere: Cluster C and D showed overlapping temporo-parietal electrodes in the time windows from 22 to 94 ms and from 256 to 316 ms, while Cluster E encompassed more occipitally located electrodes between 464 and 802 ms.

## Discussion

Processing of motor and visual abstract concepts within a deep conceptual decision task in Experiment 2 again yielded differential feature-specific ERP effects. However, unlike in Experiment 1, ERP effects already emerged at about 22 ms after target presentation. Motor abstract concepts were specifically associated with significantly more positive potentials in fronto-(central) clusters, whereas the processing of visual abstract concepts was specifically related to more positive scalp potentials in temporo-parietal and occipital electrode clusters.

Results of Experiment 2 mostly parallel results of Experiment 1, even though feature-specific ERP effects emerged earlier in Experiment 2 than in Experiment 1. The very early emergence of differential effects in Cluster C 22 ms after target presentation might reflect priming processes, in which the preceding context word preactivates certain abstract concepts leading to fast access to modal information during processing of the abstract target concept. Furthermore, this earlier onset of feature-specific effects is in line with previous work on concrete concepts demonstrating earlier feature-specific activity in modality-specific brain regions during deep conceptual tasks (Papeo et al., 2009; Popp et al., 2019; Sato et al., 2008). This early onset further suggests that visual and motor information is rapidly accessed during conceptual processing. However, since other clusters (in particular clusters B and E) covered protracted and therefore also late periods, semantic elaboration, imagery or spreading activation processes might also take place, after initial conceptual access.

Regarding the present topographic pattern, the deep conceptual decision task seems to shift effects toward the right hemisphere leading to more lateralized ERP effects in Cluster B as compared to those of Experiment 1. Comparing Cluster 1 of Experiment 1 and Cluster A of Experiment 2, which both reflect relatively early motor feature effects, Cluster A (Experiment 2) primarily includes left frontal electrodes. The deep conceptual decision task seems to shift early motor feature effects toward the left frontal scalp as seen in Cluster A, while later effects of Experiment 1 were found at more centrally located electrodes as seen in Cluster 1. Different locations of Clusters A and 1 might be the result of different neural generators within the motor cortex with Cluster A being related to more left lateralized premotor regions compared to Cluster 1. However, as already indicated above, care must be taken with regard to the localizational value of EEG data rendering the latter considerations highly speculative. Beyond that, as in Experiment 1, the polarity, topography as well as the time course of differential ERP effects of Experiment 2 are comparable to those of earlier studies examining the processing of concrete concepts (Kiefer, 2001, 2005; Martin et al., 2006; Sim & Kiefer, 2005; Trumpp et al., 2014). Taken together, results of Experiment 2 suggest i) that motor and visual abstract concepts are processed in different neural circuits (see Experiment 1), and ii) that the deep conceptual decision task enhanced feature-specific processing of abstract concepts as indicated by earlier feature-specific effects as compared to Experiment 1.

## General Discussion

The present work investigated the time course of the semantic processing of motor and visual abstract concepts using ERPs. Firstly, we asked whether feature-specific ERP effects for motor and visual abstract concepts would be similarly observed as for concrete concepts. Secondly, we assessed whether possible differential ERP effects would emerge in early (between 150 and 300 ms) or in later time windows. Such early ERP effects most likely reflect semantic access and not post-conceptual processing. Thirdly, we tested whether processing of abstract concepts is prone to conceptual flexibility and assessed whether a deep conceptual decision task leads to earlier feature-specific ERP effects compared to a shallow lexical decision task, similar to observations in concrete concepts.

ERP analyses of Experiment 1, in which participants had to perform a shallow lexical decision task, revealed feature-specific effects in fronto-central, parieto-occipital and fronto-temporal scalp regions emerging 178 ms after target onset and extending to later time windows until 680 ms. Experiment 2, a deep conceptual decision task, yielded differential scalp potentials in fronto-central, temporo-parietal and occipital electrode clusters 22 ms after target onset, with some of these clusters covering protracted and therefore also late periods.

The behavioral data of both experiments yielded comparable mean ERs (Experiment 1 & 2) and mean RTs (Experiment 2) for motor and visual abstract concepts, thus paralleling results of our earlier pilot studies (Harpaintner et al., 2020). The comparable behavioral data pattern for both subcategories of abstract concepts rules out the possibility that differential scalp potentials were due to differences in task difficulty. In a similar vein, the comparable high mean numbers of artifact-free EEG segments for motor and visual abstract concepts in Experiment 1 and 2 ensure that differences in ERPs were not due to differences in signal-to-noise ratios across conditions.

### Topography of feature-specific effects

Regarding the present electrophysiological results of Experiment 1, ERPs in response to motor and visual abstract concepts showed differential polarity patterns in fronto-central, parieto-occipital and fronto-temporal scalp regions. Paralleling the topographic results of the first experiment, Experiment 2, a deep conceptual decision task, yielded differential feature-specific ERP effects in fronto-central, temporo-parietal and occipital electrode clusters. However, comparing the first and the second Experiment, it is noticeable that Cluster B of Experiment 2 shows more lateralized ERP effects than the first experiment. The underlying causes must remain speculative, but the deep conceptual decision task seems to shift effects toward the right hemisphere. Different topographic patterns of Cluster 1 of Experiment 1 and Cluster A of Experiment 2, with Cluster A being located more left lateralized at frontal electrodes, might further reflect different neural generators within the motor cortex for the two clusters. Even though the underlying causes, here too, must remain speculative, the deep conceptual decision task seems to shift effects of Cluster A to more left lateralized premotor regions as compared to Cluster 1.

The feature-specific ERP effects in response to motor and visual abstract concepts are largely comparable to earlier EEG studies, which investigated the processing of concrete concepts: Similar to concrete motor concepts (Hauk & Pulvermüller, 2004; Kiefer, 2001, 2005; Popp et al., 2016; Pulvermüller et al., 1999, 1996; Trumpp et al., 2014), abstract motor concepts were associated with relatively more positive potentials over frontal and central scalp regions, whereas abstract visual concepts, similar to concrete visual concepts (Kiefer, 2001, 2005; Martin et al., 2006; Pulvermüller et al., 1999, 1996; Sim & Kiefer, 2005), were specifically related to more positive potentials over parieto-occipital as well as over temporal scalp regions. Although the spatial resolution provided by the EEG must be interpreted with caution (Nunez, 1981), the topography of the present feature-specific effects is nevertheless in line with findings of a previous neuroimaging study using the same set of abstract concepts as stimuli (Harpaintner et al., 2020). This fMRI study revealed that the processing of motor abstract concepts was associated with an enhanced BOLD signal in frontal and parietal motor regions, similarly as the execution of real movements. The processing of visual abstract concepts, instead, was related to enhanced activity in temporal and occipital visual brain areas, similar as the observation of object pictures. Furthermore, numerous fMRI studies on concrete concepts linked the processing of motor concepts to an increased activity in fronto-central motor regions (Hauk, Johnsrude, & Pulvermüller, 2004; Hauk & Pulvermuller, 2011; Kemmerer, Castillo, Talavage, Patterson, & Wiley, 2008; Pulvermüller, Cook, & Hauk, 2012; Pulvermüller, Kherif, Hauk, Mohr, & Nimmo-Smith, 2009; Raposo, Moss, Stamatakis, & Tyler, 2009; Rüschemeyer, Brass, & Friederici, 2007; Tomasino, Weiss, & Fink, 2010; Willems, Hagoort, & Casasanto, 2010), whereas the processing of visual concepts was associated with an increased activity in the occipital and temporal lobe (Devlin et al., 2002; Perani et al., 1995; Simmons et al., 2007).

Whereas the topography of the rather late clusters of Experiment 1 comparing visual vs. motor abstract concepts is highly compatible with previous electrophysiological and neuroimaging findings, Cluster 5 showing differential feature-specific ERPs in right fronto-temporal electrodes requires detailed consideration. As already discussed above, this topographic difference compared to findings on concrete concepts might be due to differential conceptual representations of concrete vs. abstract concepts or might reflect interindividual neuroanatomical differences of samples. Based on findings of our previous fMRI study (Harpaintner et al., 2020), the fronto-temporal electrode cluster might also reflect activity in the anterior part of the fusiform gyrus. Referring to recent hybrid models (Kiefer & Pulvermüller, 2012; Patterson et al., 2007), Cluster 5 might also be based on an increased activity in the temporal pole, which has been considered a prominent candidate for a hub region (Patterson et al., 2007), although our previous neuroimaging study did not reveal differential activity in this region (Harpaintner et al., 2020). Finally, the fronto-temporal electrode cluster might be the result of a paradoxical localization based on the direction of the electrical current flow originating from the left fusiform gyrus, a phenomenon characteristically obtained with regard to the N400 component. Even though the maximum of the N400 is typically observed at right parieto-central electrodes, its neural sources constantly trace back to the left fusiform gyrus and the left medial temporal lobe (Kutas & Federmeier, 2011). A similar mechanism might also have taken place with regard to Cluster 5.

### Time course of feature-specific effects

Cluster permutation tests revealed significant differences between the processing of motor vs. visual abstract concepts emerging 178 ms after target onset for Experiment 1. The start of differential ERP effects was earlier in Experiment 2, demonstrated by Cluster C, in which modality-specific effects emerged 22 ms after target onset. As already discussed above, this very early emergence of differential effects in Cluster C might be a result of priming processes, caused by the context word preceding the abstract target word. Furthermore, the earlier onset of differential effects in Experiment 2, in which a retrieval of conceptual information is demanded, is in line with previous work on concrete concepts demonstrating earlier feature-specific activity in modality-specific brain regions during deep conceptual tasks (Papeo et al., 2009; Popp et al., 2019; Sato et al., 2008). Most importantly, the differential onsets of the first and second experiment speak in favor of conceptual flexibility by showing that ERP effects were modulated by task.

Earlier studies (Adorni & Proverbio, 2012; Barber et al., 2013; Bardolph & Coulson, 2014; Palazova et al., 2013; West & Holcomb, 2000; Wirth et al., 2008), which examined the time course of the processing of abstract concepts, were particularly limited to the comparison of concrete vs. abstract concepts. A key finding of these studies was that specific electrophysiological effects, like the emotion related early posterior negativity effect (Palazova et al., 2013) or congruency effects (Bardolph & Coulson, 2014), occur later in abstract concepts than in concrete concepts (Borghi et al., 2017). This result pattern led some researchers to conclude that ERP effects reflect mental imagery instead of lexico-semantic processes (Adorni & Proverbio, 2012; Barber et al., 2013; Borghi et al., 2017). As outlined in the introduction section, late ERP effects might reflect post-conceptual imagery processes and therefore do not preclude the existence of amodal conceptual representations, which are accessed earlier. For that reason, only demonstration of early sensorimotor activity during a conceptual task, reflecting access to conceptual representations rather than post-conceptual processes, can be taken as unequivocal evidence for grounded cognition theories (Kiefer & Pulvermüller, 2012).

However, abstract and concrete concepts differ with regard to a variety of variables such as familiarity, word frequency or age of acquisition, rendering a direct comparison difficult. Furthermore, as discussed in the introductory section, abstract concepts are highly heterogeneous with regard to their semantic content and should not be considered as uniform conceptual category (Kiefer & Harpaintner, 2020). Making use of a theory-driven approach and comparing electrophysiological responses to specific subgroups of abstract concepts with a known feature composition, we showed that abstract concepts are associated with both, early and relatively late feature-specific ERPs. The early emergence of differential scalp potentials in both Experiments 22 and 178 ms after target onset, dependent on the task, suggests that effects reflect rapid conceptual access of motor and visual information. After initial conceptual access, however, post-conceptual processes such as imagery, semantic elaboration or spreading activation might take place, as indicated by the subsequent clusters showing feature-specific ERPs in later time windows.

### Implications for theories of conceptual representations, limitations and further directions

The findings of Experiment 1 and 2 are difficult to reconcile with traditional amodal theories of conceptual representation, which assume the representational format of concepts, especially of abstract concepts, to be independent of original modality-specific experiential information (Anderson, 1978; Collins & Loftus, 1975; Fodor, 2001; Mahon & Caramazza, 2009; Pylyshyn, 1980). The early onset of feature-specific ERPs furthermore invalidates the argument of amodal theories that differential effects, often found in brain imaging studies, explicitly rely on later imagery or elaborative processes or spreading activation. Instead, our results suggest distinct feature-specific conceptual processing circuits for abstract concepts implemented by the grounding of conceptual representations in perception and action. In line with grounded cognition theories (Barsalou, 2008; Borghi et al., 2017; Ghio et al., 2016; Kiefer & Barsalou, 2013; Kiefer & Harpaintner, 2020; Pulvermüller & Fadiga, 2010), our findings indicate that motor and visual abstract concepts, similar to concrete concepts, are processed in distinct brain areas (see also Pulvermüller & Henningsen, this issue). Together with our earlier fMRI study (Harpaintner et al., 2020), which provides precise anatomical information for the presently observed feature-specific effects, the results of this ERP study suggest that abstract concepts are represented in modality-specific sensorimotor brain areas, even if they lack a clear physical referent. Our results furthermore support theoretical considerations of grounded cognition theories that access to conceptual knowledge is highly flexible (Kiefer & Pulvermüller, 2012; Kuhnke et al., 2020) by showing that feature-specific ERPs are differentially affected by task demands in Experiment 1 and 2. Overall, the present results are consistent with hybrid theories of conceptual representation proposing that conceptual knowledge is based on an interaction between modality-specific, multimodal and amodal conceptual hub areas (Fernandino et al., 2016a; Fernandino, Humphries, Conant, Seidenberg, & Binder, 2016b; Garagnani & Pulvermüller, 2016; Harpaintner et al., 2020; Kiefer & Harpaintner, 2020; Kuhnke et al., 2020; Popp et al., 2019; Simmons & Barsalou, 2003).

As EEG studies only provide correlational data, methods that render causal conclusions possible are inevitable in order to show that sensorimotor information is necessary for the processing of abstract concepts. Further work making use of transcranial magnetic stimulation (Pulvermüller et al., 2005a; Vukovic, Feurra, Shpektor, Myachykov, & Shtyrov, 2017), behavioral interference paradigms (Shebani & Pulvermüller, 2013; Vermeulen, Corneille, & Niedenthal, 2008) or investigating brain-lesioned patients (Dreyer et al., 2015; Neininger & Pulvermüller, 2003; Trumpp et al., 2013a) seems mandatory in order to shed light on the functional relevance of modality-specific representations for the processing of abstract concepts.

It is noteworthy that we only investigated the processing of motor and visual abstract concepts, even though the feature composition of abstract concepts seems to be much richer and highly heterogeneous (Barsalou & Wiemer-Hastings, 2005; Binder et al., 2016; Harpaintner et al., 2018; Kiefer & Harpaintner, 2020; Lynott & Connell, 2009, 2013; Muraki et al., this issue; Troche et al., 2014; Troche et al., 2017; van Dantzig et al., 2011). Future work should examine abstract concepts, which are characterized by different modal features, in order to complete the picture. It is likely that other subgroups of abstract concepts, like abstract concepts with a strong link to emotions, social constellations, mental states or verbal associations, elicit ERPs with other topographies, polarities and time courses as compared to the present study.

In conclusion, the results of the present ERP experiments demonstrate differential ERP effects for motor and visual abstract concepts, whose topography parallels ERP effects of concrete motor and visual concepts (Hauk & Pulvermüller, 2004; Kiefer, 2001, 2005; Martin et al., 2006; Popp et al., 2016; Pulvermüller et al., 1999, 1996; Sim & Kiefer, 2005; Trumpp et al., 2014). A previous fMRI study (Harpaintner et al., 2020) with the same stimuli localized the neural sources of these feature-specific effects in corresponding modality-specific brain areas. Most importantly, the present study extends these earlier findings by providing information about the time course of abstract concept processing. Both the shallow lexical decision task and the deep conceptual decision task were associated with feature-specific ERPs in relatively late time windows indicating that post-conceptual processes such as imagery, semantic elaboration or spreading activation might be involved in the processing of abstract concepts. However, the emergence of differential scalp potentials before 300 ms with effects as early as 22 and 178 ms after target onset favors the assumption of grounded cognition theories that motor and visual information is also rapidly accessed in corresponding modal brain regions during conceptual processing. The fact that differential ERPs occurred earlier in the deep as compared to the shallow task furthermore indicates that the processing of abstract concepts is prone to conceptual flexibility supporting another important notion of the grounded cognition framework.

## Footnotes

1 Obtained from the University of Tübingen. Freely available under https://www.lingexp.uni-tuebingen.de/z2/LSAspaces/
